# Virus-derived DNA drives mosquito vector tolerance to arboviral infection

**DOI:** 10.1038/ncomms12410

**Published:** 2016-09-01

**Authors:** Bertsy Goic, Kenneth A. Stapleford, Lionel Frangeul, Aurélien J. Doucet, Valérie Gausson, Hervé Blanc, Nidia Schemmel-Jofre, Gael Cristofari, Louis Lambrechts, Marco Vignuzzi, Maria-Carla Saleh

**Affiliations:** 1 Institut Pasteur, Viruses and RNA Interference Unit, Centre National de la Recherche Scientifique UMR 3569, 75724 Paris cedex 15, France; 2 Institut Pasteur, Viral Populations and Pathogenesis Unit, Centre National de la Recherche Scientifique UMR 3569, 75724 Paris cedex 15, France; 3 IRCAN, INSERM U1081, Centre National de la Recherche Scientifique UMR 7284, University of Nice—Sophia-Antipolis, 06107 Nice Cedex 2, France; 4 FHU OncoAge, University of Nice-Sophia Antipolis, 06107 Nice, France; 5 Institut Pasteur, Insect-Virus Interactions Group, Centre National de la Recherche Scientifique URA 3012, 75724 Paris cedex 15, France

## Abstract

Mosquitoes develop long-lasting viral infections without substantial deleterious effects, despite high viral loads. This makes mosquitoes efficient vectors for emerging viral diseases with enormous burden on public health. How mosquitoes resist and/or tolerate these viruses is poorly understood. Here we show that two species of *Aedes* mosquitoes infected with two arboviruses from distinct families (dengue or chikungunya) generate a viral-derived DNA (vDNA) that is essential for mosquito survival and viral tolerance. Inhibition of vDNA formation leads to extreme susceptibility to viral infections, reduction of viral small RNAs due to an impaired immune response, and loss of viral tolerance. Our results highlight an essential role of vDNA in viral tolerance that allows mosquito survival and thus may be important for arbovirus dissemination and transmission. Elucidating the mechanisms of mosquito tolerance to arbovirus infection paves the way to conceptualize new antivectorial strategies to selectively eliminate arbovirus-infected mosquitoes.

## Abstract (web summary)

Mosquitoes tolerate long-lasting viral infections, which makes them efficient vectors for human viral diseases. Here, the authors show that the tolerance of *Aedes* mosquitoes to infection with dengue or chikungunya viruses depends on the production of viral-derived DNA by cellular reverse transcriptases.

## Graphical abstract

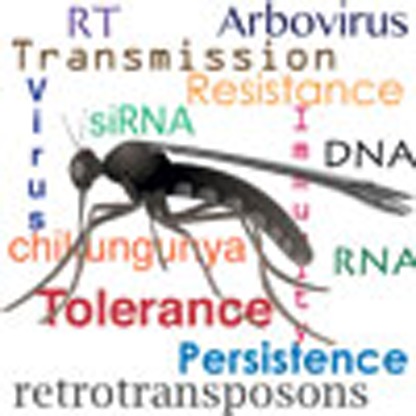

Arthropods play an essential role in global ecosystems and in the development of agricultural economies. However, some of them are capable of spreading severe pathogens to humans, livestock and crops resulting in devastating consequences. Among these, mosquitoes cause hundreds of millions of infections every year^1^, as they are vectors for a wide variety of pathogens including malaria parasites and arboviruses (arthropod-borne viruses) such as dengue, Zika and chikungunya (CHIKV) viruses. Despite their impact, little is known about the mechanisms by which mosquitoes are able to carry and transmit viral pathogens.

Currently, the majority of our knowledge on insect antiviral immune responses comes from studies in *Drosophila melanogaster*^2^ and more limited work in mosquitoes^3^,^4^,^5^,^6^,^7^,^8^. These reports have revealed the essential role of RNA interference (RNAi) pathways in antiviral immune responses, which include the small interfering RNA (siRNA) pathway in both *Drosophila* and mosquitoes, as well as the contribution of the piwi-interacting RNA (piRNA) pathway solely in mosquitoes^6^,^9^,^10^,^11^,^12^. To elicit an antiviral response, the siRNA pathway is triggered by double-stranded RNA (dsRNA) molecules from viral genomes and replicative intermediates. These pathogen-associated molecular patterns are recognized and cleaved by Dicer-2 (Dcr-2) into 21 nts viral siRNAs (vsiRNA). Once produced, vsiRNAs guide the sequence-specific recognition and cleavage of viral RNAs by Argonaute-2 (ref. 13). On the other hand, piRNAs range in size between 26 and 31 nts with a bias for a 5′ uridine in both vertebrates and invertebrates^14^,^15^. Although they have been mostly linked to epigenetic and post-transcriptional silencing of retrotransposons and other genetic elements in the germ line, some studies have also suggested an antiviral role in mosquito somatic cells^6^,^8^,^9^,^12^,^16^.

While these antiviral pathways help to control infections in insects, they do not eliminate viral pathogens, resulting in a long-lasting viral infection or the so-called viral persistent infection with minor fitness costs for the host. Such a situation of low virulence and the ability to buffer the negative impacts on host fitness, despite high pathogen load, has been described as a defense strategy called tolerance^17^,^18^. Tolerance diverts fewer resources from the immune response and minimizes the resulting self-inflicted damages. Thus, immune tolerance is an adaptive strategy in terms of survival to a recurrent pathogen and its associated damage^19^. In contrast, a strategy called resistance, involves the activation of immune pathways that target pathogens to control their replication. Resistance avoids infection, reduces pathogen load and eventually results in pathogen clearance^20^,^21^. Nevertheless, successful clearance through resistance is often costly in terms of energy and resources^20^,^22^. Both tolerance and resistance rely on sensing mechanisms and on a threshold of responsiveness. A prevailing model postulates that danger signals are required to activate an appropriate defense against pathogens, which could be released by damaged infected tissues, and that the levels of these signals should correlate with a damage threshold^19^,^23^,^24^,^25^.

Recently, we showed that flies infected with RNA viruses produce viral-derived DNA (vDNA) molecules through the activity of endogenous retrotransposons, a cellular source of reverse transcriptase activity. These vDNA molecules boost the RNAi-mediated antiviral immune response and are indispensable for establishing persistent viral infections in *Drosophila melanogaster*^26^. Since arboviruses are nearly all RNA viruses, while belonging to a wide range of viral families, we hypothesized that vDNA synthesis could also impact arboviral dissemination and transmission, and thus could be a general phenomenon involved in viral defense in arthropods. Notably, it could provide a molecular basis to explain how mosquitoes maintain persistent infections to become efficient vectors of viral pathogens.

Here we show that arbovirus-infected mosquitoes produce vDNA forms, which are essential for the insect's survival. When vDNA production is inhibited, the levels of virus-specific small RNA are reduced and mosquitoes die from a loss of tolerance towards viral infection. We speculate that these vDNA molecules might act as a danger signal to reach and maintain a state of tolerance response, leading to persistent infection in the host.

## Results

### Mosquito cells produce arbovirus-derived DNA *in vitro*

**Figure 1:**
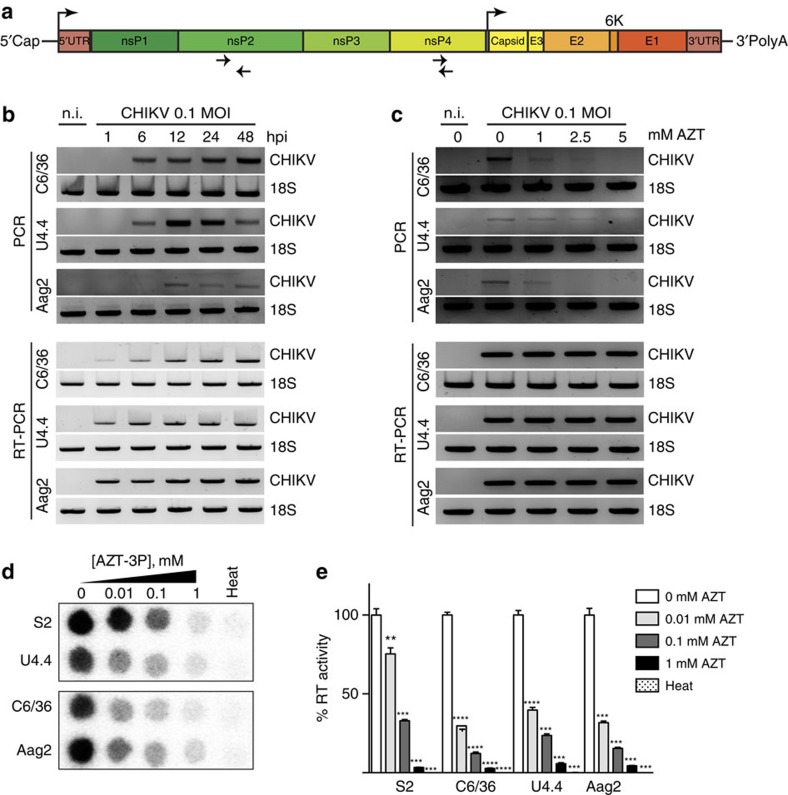
Mosquito cells produce host reverse transcriptase-dependent arbovirus-derived DNA. (**a**) Schematic of CHIKV viral genome. Top arrows indicate the position of the genomic and subgenomic promoters. Bottom arrows indicate the position of the primers used for vDNA detection. (**b**) Kinetics of vDNA synthesis. C6/36, U4.4 and Aag2 cells were infected with CHIKV at a MOI of 0.1 and cells were harvested at the indicated time points. Cells were analysed by PCR (upper panel) for vDNA detection. RT-PCR (lower panel) was used to follow viral infections. Non-infected cells (n.i) were used as a negative control and cellular 18S rRNA was used as a housekeeping gene loading control. (**c**) AZT inhibits vDNA synthesis *in vitro*. C6/36, U4.4 and Aag2 cells were treated with increasing concentration of AZT for 2 days. At the indicated time point, cells were harvested and vDNA and RNA were assessed as described in **b**. (**d**–**e**) Endogenous reverse transcriptase activity in insect cells. (**d**) Dose-dependent AZT inhibition was tested in insect cell extracts and (**e**) quantification of reverse transcriptase inhibition expressed as an activity percentage of non-treated extracts. Heat inactivated samples (heat) were used as negative controls. *Drosophila* S2 cells were used as a positive control. Each experiment was completed at least 3 times. Error bars correspond to the s.d. *t*-test with Welch's correction was used to determine statistical significance compared with the untreated control as a reference (**P<0.01; ***P<0.001; ****P<0.0001).

We previously identified a link between vDNA molecules and viral persistence in the insect model *Drosophila melanogaster*^26^. However, the presence of similar mechanisms in other arthropods, including vectors of human disease such as mosquitoes, is unknown. To explore this possibility, we used the arbovirus CHIKV from the Indian Ocean lineage, and its mosquito vectors *Aedes albopictus* and *Aedes aegypti*. To assess the presence of CHIKV-specific vDNA, we first optimized PCR and primer conditions to minimize and remove nonspecific amplification (data not shown). Using multiple primer pairs spanning the entire CHIKV genome, we identified two primer pairs that generated amplicons of roughly 600 and 200 bp in the nonstructural proteins (nSP) 2 and 4, respectively, that had no cross-reactivity with uninfected cells (Fig. 1a). As this PCR approach specifically amplifies two regions of the virus as vDNA, we cannot exclude that other vDNA segments might also be synthesized, but not detected by our assay. In *D. melanogaster*, vDNA is highly rearranged compared with its vRNA counterpart^26^. This phenomenon might explain why by conventional PCR we did not detect more vDNA species. We then infected *Ae. albopictus* and *Ae. aegypti* cell lines with CHIKV and looked for the presence of vDNA by virus-specific PCR. We detected vDNA in all cell lines tested (Fig. 1b) and a kinetic analysis of vDNA synthesis revealed that it can be detected as early as 6 h after infection in *Ae. albopictus* cell lines (C6/36 and U4.4 cells) and 12 h after infection in *Ae. aegypti* cells (Aag2) (Fig. 1b, full gels available in Supplementary Fig. 9).

Cellular reverse transcriptase activity originating from endogenous retrotransposons is responsible for vDNA formation in *Drosophila*^26^. To test if this could also be the case in mosquito species, and to get insight into the mechanism of vDNA formation, we first treated mosquito cell lines with azidothymidine (AZT), a reverse transcriptase inhibitor previously shown to inhibit the synthesis of vDNA in *Drosophila*^26^. Indeed, addition of AZT to the culture medium at the time of infection prevents CHIKV vDNA accumulation in a dose-dependent manner, with complete inhibition reached at 5 mM AZT (Fig. 1c, full gels available in Supplementary Fig. 9). Importantly, AZT treatment does not reduce viral titres nor viral RNA synthesis, suggesting that this drug does not interfere with viral RNA replication (Supplementary Fig. 1a,b). Finally, given the function of AZT as a reverse transcriptase inhibitor, we hypothesized that cellular reverse transcriptase activity is playing a major mechanistic role in vDNA synthesis. Indeed, we could detect endogenous reverse transcriptase activity in non-infected mosquito cell extracts (Supplementary Fig. 1c); and this activity is sensitive to AZT-triphosphate (AZTTP) *in vitro* (Fig. 1d,e). Taken together, these data show that *Ae. albopictus* and *Ae. aegypti* cells can generate CHIKV-specific vDNA and that its synthesis is dependent on a cellular reverse transcriptase activity.

**Figure 2:**
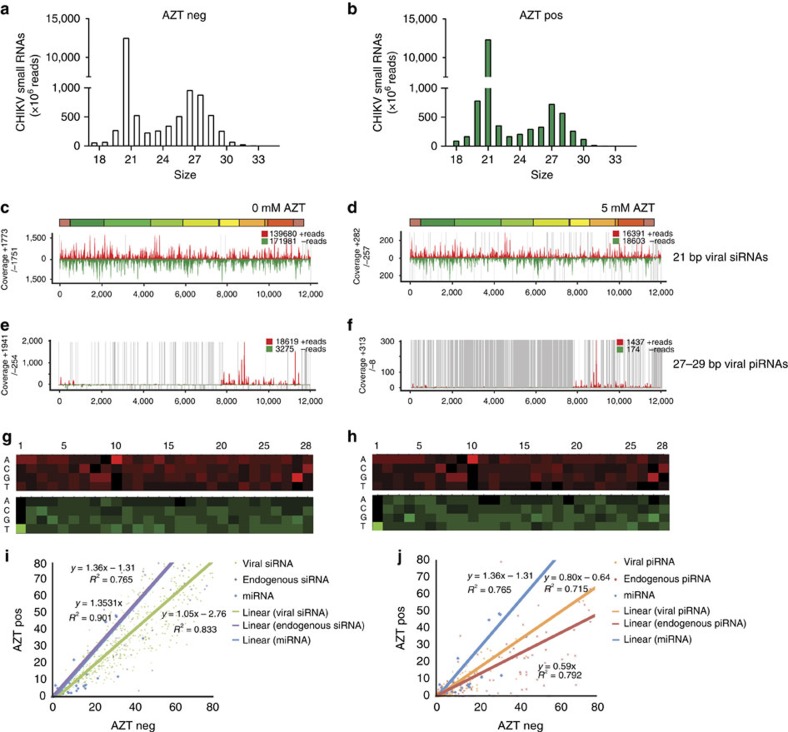
Chikungunya vDNA is required for small viral RNA production in cultured cells. vDNA contributes to the production of vpiRNAs in mosquito cell lines. Infected U4.4 cells were (**a**) left untreated (clear bars) or (**b**) treated with 5 mM AZT (green bars) for 3 days. Graph represents the size distribution of the total number of CHIKV-specific small RNA reads (ranging from 18 to 33 nts) normalized to the total number of reads. (**c**) vsiRNAs in U4.4 cells show homogenous coverage over the entire genome. (**d**) vsiRNA coverage in AZT-treated cells. Uncovered regions are represented as grey lines. The coverage of vpiRNAs on the CHIKV genome in (**e**) untreated cells or (**f**) AZT-treated cells. Most of the CHIKV vpiRNA reads belong to the 3′-terminal region on the viral genome. The sense and anti-sense small RNAs are in red and green, respectively. (**g**-**h**) Relative nucleotide frequency per position of the 27–29 nt viral small RNAs that map to the sense and anti-sense strand of the viral genome, red and green respectively. The intensity varied in correlation with the frequency. A nucleotide bias (U1 and A10) is observed. (**i**,**j**) Accumulation of viral and cellular small RNAs in U4.4 cells 3 days post CHIKV infection in the presence (AZT pos) or absence (AZT neg) of AZT, assessed as (**i**) mapping of small RNAs corresponding to CHIKV vsiRNA (green), endo-siRNA (purple) belonging to the gene *GAPW01000199* or to miRNA (blue). For miRNA each dot represents one miRNA. For siRNAs each dot represents the coverage of a region of 20 bases of the target RNA. The lines for miRNA and endo-siRNA are superposed. (**j**) Mapping of small RNA corresponding to CHIKV vpiRNA (orange), endo-piRNA (red) belonging to the gene *GAPW01000199* or to miRNA (blue). For miRNAs each dot represents one miRNA. For piRNAs each dot represents the coverage of a region of 20 bases of the target RNA. For (**i**,**j**) lines represent the linear trendline of each set of values. The equation and *R*^2^ value of each regression are also mentioned.

To determine whether vDNA has an effect on the cellular immune response, we generated small RNA libraries from *Ae. albopictus* cells 3 days after CHIKV infection. As previously described^27^, C6/36 cells mainly produce viral-specific piRNAs (vpiRNAs; 27–29 nts, Supplementary Fig. 2a–f) with a ping-pong signature, indicative of an amplification process, and a U1 bias for anti-sense small RNAs and an enrichment of A10 in sense small RNAs (Supplementary Fig. 2g,h). AZT treatment of this cell line did not result in changes in vsiRNAs or vpiRNAs (Supplementary Fig. 2i,j). In U4.4 cells, we observed the generation of both vpiRNAs and viral-specific siRNAs (vsiRNAs), during CHIKV infection (Fig. 2a,b)^6^. In addition, we found that vpiRNAs are mainly generated from the subgenomic region of the CHIKV genome, whereas vsiRNAs align over the entire CHIKV genome (Fig. 2c–f). Interestingly, when U4.4 cells were treated with AZT to inhibit vDNA synthesis, we did not observe major changes in the levels of endogenous or vsiRNAs (Fig. 2i). However, we observed a decrease in the amount of endogenous piRNAs and vpiRNAs with no change of the endogenous microRNAs (miRNA), indicating that AZT treatment is interfering with piRNA production in cultured cells (Fig. 2j). Although AZT does not interfere with vsiRNA production in U4.4 cells, it could still interfere with the siRNA pathway, downstream of vsiRNAs production, for example, by reducing its silencing activity. To test this possibility, we employed an *in vitro* luciferase-silencing assay in the absence or presence of AZT. We found that AZT treatment of mosquito cells had no effect on silencing, indicating that the RNAi pathway remains functional (Supplementary Fig. 3). These results suggest that, in cultured mosquito cells, reverse-transcriptase-mediated vDNA formation can influence viral small RNA production, particularly vpiRNAs, but does not seem to impact vsiRNA production or function.

### vDNA is required for viral small RNA production *in vivo*

**Figure 3:**
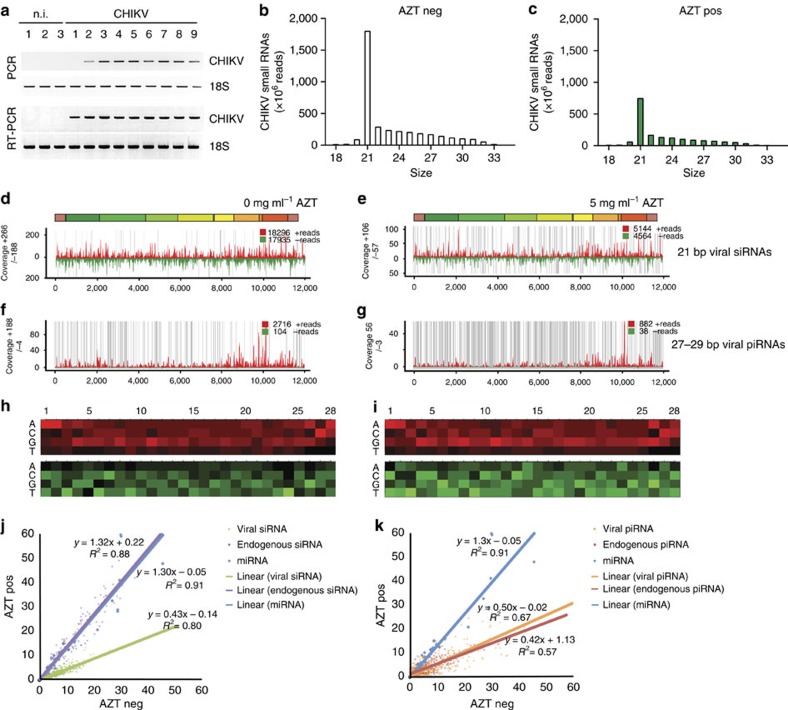
Arbovirus-infected mosquitoes produce vDNA that contributes to the production of vsiRNAs *in vivo*. (**a**) *Ae. albopictus* mosquitoes were infected with CHIKV and analysed for vDNA 9 days post infection. Three non-infected (n.i.) and nine CHIKV-infected mosquitoes were tested. 18S rRNA was used as a control. (**b**) Mosquitoes were untreated (clear bars) or (**c**) treated with 5 mg ml^−1^ AZT (green bars) and harvested 3 dpi. Five whole mosquitoes were pooled from each condition for the generation of small RNA libraries. Graphs represent the size distribution of the total number of CHIKV-specific small RNA reads (corresponding to the positive and negative strand orientation of the viral genome) ranging from 18 to 33 nts normalized by the total number of reads. (**d**–**g**) Panels show the coverage of CHIKV genome at 3 dpi using the 21 or 27–29 nts-long small RNAs at the different conditions (untreated: **d** and **f**, or AZT-treated: **e** and **g**). The sense and anti-sense small RNAs are in red and green, respectively. Grey lines represent uncovered regions. (**h**,**i**) Relative nucleotide frequency per position of the 27–29 nt viral small RNAs that map to the sense and anti-sense strand of the viral genome, red and green respectively. The intensity varied in correlation with the frequency. No nucleotide bias (U1 and A10) is observed. (**j**,**k**) Accumulation of viral and cellular small RNAs in *Ae. albopictus* 3 days post CHIKV infection in the presence (AZT pos) or absence (AZT neg) of AZT, assessed as (**j**) mapping of small RNAs corresponding to CHIKV vsiRNA (green), endo-siRNA (purple) belonging to the gene *GAPW01000199* or to miRNA (blue). The lines for miRNA and endo-siRNA are superposed. (**k**) Mapping of small RNA corresponding to CHIKV vpiRNA (orange), endo-piRNA (red) belonging to the gene *GAPW01000199* or to miRNA (blue). For (**j**,**k**) each blue dot represents one miRNA. For siRNAs and piRNAs each dot represents the coverage of a region of 20 bases of the target RNA. Lines represent the linear trendline of each set of values. The equation and *R*^2^ value of each regression are also mentioned.

Given the presence of CHIKV vDNA in infected mosquito cells *in vitro*, we next addressed whether vDNA can also form *in vivo*. We infected *Ae. albopictus* mosquitoes with CHIKV, allowed the infection to develop for 9 days (during the peak of viral replication), and then looked for the presence of vDNA. CHIKV vDNA was detected in all infected mosquitoes, whereas non-infected individuals were negative for both CHIKV vDNA and RNA (Fig. 3a, full gels available in Supplementary Fig. 10), indicating that vDNA is not only generated in cultured cells but also *in vivo*. We also searched for vDNA generated on infection of *Ae. aegypti* with dengue virus and, as for CHIKV, we readily detected vDNA (Supplementary Fig. 4), suggesting that the presence of vDNA is a common feature of arboviral infections found in mosquitoes from different species (*Ae. albopictus* and *Ae. aegypti,* respectively) harbouring arboviruses from different families (*Togaviridae* and *Flaviviridae* for CHIKV and dengue virus, respectively).

To address whether vDNA contributes to the RNAi response *in vivo*, we looked at the small RNA profiles of mosquitoes at 3 days (early, Fig. 3b–k) and 9 days (late, Supplementary Fig. 5) post-infection, with or without AZT treatment. To do this, we allowed female mosquitoes to feed for 2 days on sucrose alone or sucrose supplemented with 5 or 10 mg ml^−1^ of AZT to suppress endogenous reverse transcriptase activity before infection. Mosquitoes were then infected with CHIKV via artificial blood meal and AZT was supplied daily throughout the duration of the experiment. Interestingly, and in contrast to what we observed in cultured cells, in the absence of AZT (and thus in the presence of vDNA), the majority of virus-specific small RNAs were vsiRNAs (Fig. 3b) and the small fraction of detected vpiRNA-like molecules lacked a ping-pong signature (Fig. 3h). Both types of small RNAs span the entire CHIKV genome sequence (Fig. 3d-g). On AZT treatment, we observed a decrease of vsiRNA and vpiRNA-like levels in mosquitoes at day 3 post-infection, consistent with a role for vDNA in the production of vsiRNAs and possibly vpiRNA precursors *in vivo* (Fig. 3j,k). These changes of viral small RNA levels were not accompanied by alterations of the size distribution of these RNAs (Fig. 3c), nor by a change in the global levels of endogenous miRNAs or siRNAs (Fig. 3j-k), indicating that AZT does not interfere in general with the RNAi machinery *in vivo*. When we analysed viral small RNAs at a later stage of the infection (9 days post-infection), we detected increased levels of vsiRNA in both AZT-treated and -untreated mosquitoes, as compared with earlier stage, as well as the production of vpiRNAs presenting now a ping-pong signature (canonical vpiRNAs; Supplementary Fig. 5). As observed in cultured cells, vsiRNA reads cover the entire CHIKV genome where vpiRNA reads align to the subgenomic region. When we analysed the viral small RNA response at day 3 post-infection in mosquitoes treated with 10 mg ml^−1^ AZT, we found an even stronger reduction of vsiRNAs (Supplementary Fig. 6), confirming the role of vDNA in the early phase of viral small RNAs production. These results indicate that, *in vivo*, mosquitoes use a siRNA-specific response to control and maintain CHIKV infection and that early vsiRNA population is dependent on vDNA synthesis.

### vDNA is essential for mosquito tolerance to arbovirus infection

**Figure 4:**
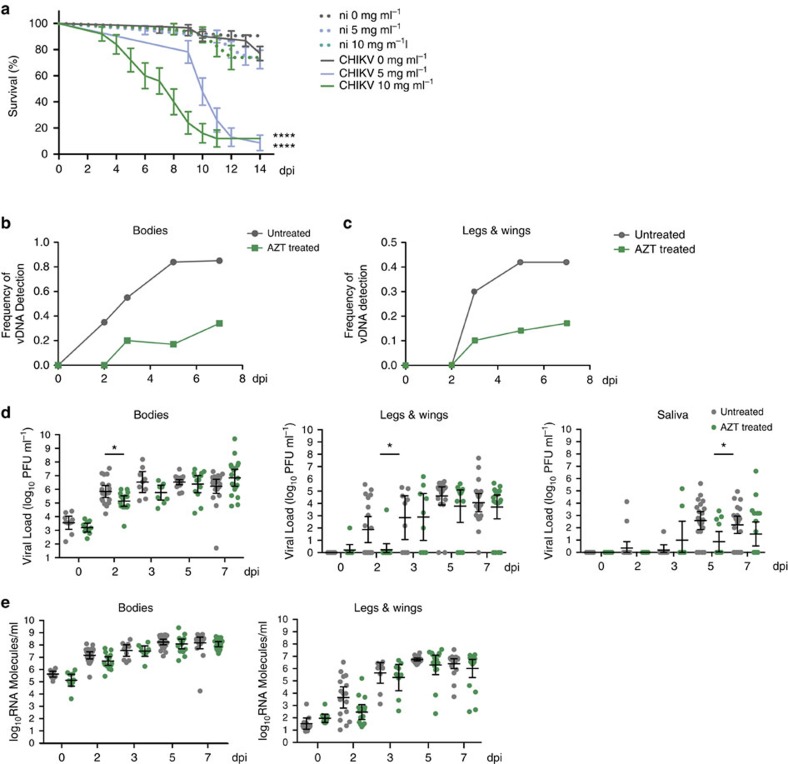
vDNA is required for mosquito tolerance to CHIKV infection. (**a**) Survival curve of CHIKV-infected mosquitoes treated daily with 0, 5 or 10 mg ml^−1^ of AZT. After the infectious blood meal survival of mosquitoes was monitored daily for 14 days. Continuous lines show the lifespan of AZT-treated and infected mosquitoes while dotted lines show lifespan of AZT-treated but non-infected (n.i.) mosquitoes. Grey lines: 0 mg ml^−1^ AZT; blue lines: 5 mg ml^−1^ AZT and green lines: 10 mg ml^−1^ AZT. At least 40 mosquitoes were used for each condition. *P*-values were calculated using a Gehan–Breslow–Wilcoxon test using uninfected mosquitoes with the same AZT treatment as a reference. (**b**,**c**) Frequency of vDNA in mosquitoes treated with 10 mg ml^−1^ AZT (green, squares) or untreated mosquitoes (grey, circles) in (**b**) bodies or (**c**) legs and wings. (**d**) Individual mosquito viral titres quantified by plaque assay. Viral titres were determined in bodies (left panel), legs and wings (middle panel) and saliva (right panel) for untreated mosquitoes (grey dots) or mosquitoes treated with 10 mg ml^−1^ AZT (green dots) at the indicated time points. Standard deviation and geometric mean are shown. At least 10 mosquitoes were used for each time point. Error bars represent the s.d. A Wilcoxon rank-sum test was used to determine statistical significance (*P<0.05, ****P<0.0001). Absence of *P*-value represents non-statistical significance. (**e**) Number of viral RNA molecules were calculated by single mosquito qRT-PCR in bodies (left panel) and legs and wings (right panel) for untreated mosquitoes (grey dots) or mosquitoes treated with 10 mg ml^−1^ AZT (green dots) at the indicated time points. Absence of *P*-value represents non-statistical significance. Each experiment was completed at least three times.

To address whether vDNA is related to viral maintenance by resistance or tolerance, we compared mosquito survival and viral titres between AZT-treated insects and their untreated controls on viral challenge. As expected, non-infected mosquitoes (whether untreated or AZT-treated) and infected (yet untreated) mosquitoes survived the 14 days duration period of the experiment (Fig. 4a). However, CHIKV-infected mosquitoes that were treated with 5 or 10 mg ml^−1^ of AZT died rapidly over the course of 2 weeks (Fig. 4a and Supplementary Fig. 7). In addition, we found that death rate was dose-dependent as mosquitoes treated with 10 mg ml^−1^ of AZT succumbed from infection earlier and with a steadier rate than those treated with 5 mg ml^−1^, which died rapidly starting at 9 days post-infection. These data suggest that, similar to what was seen in *Drosophila*, the presence of vDNA promotes viral persistence in the infected host, and eventually host survival.

To address the kinetics of vDNA production *in vivo*, CHIKV-infected mosquitoes were treated with 10 mg ml^−1^ of AZT as described in the previous section. Untreated mosquitoes produced and accumulated CHIKV vDNA in a temporal manner, with roughly 80% of infected mosquitoes presenting vDNA in their bodies 7 days post-infection (Fig. 4b). However, we observed a significant delay and reduction in the production of vDNA in AZT-treated mosquitoes, with only 35% of mosquitoes presenting vDNA in their bodies 7 days post-infection (Fig. 4b). Interestingly, we also detected the presence of vDNA in legs and wings, a possible evidence for circulating and disseminating vDNA, and again found a decrease in the frequency of vDNA in AZT-treated mosquitoes (Fig. 4c).

We then addressed whether the cause of death was associated to a loss of resistance or tolerance to the viral infection. Given the rapid death of infected mosquitoes in the absence of vDNA, we hypothesized that the mosquitoes were dying from an overwhelming viral burden or loss of resistance. We quantified viral titres at each time point in untreated and AZT-treated mosquitoes. The untreated mosquitoes displayed typical kinetics of CHIKV replication, with viral titres peaking in the bodies at day 3 post-infection, and dissemination and transmission beginning at day 2 post-infection (Fig. 4d, and Supplementary Fig. 8, grey dots). Interestingly, in the absence of vDNA we observed a delay in viral infection (bodies) and dissemination (legs and wings) at day 2, and in transmission potential (saliva) at day 5 (Fig. 4d and Supplementary Fig. 8, green dots). One possibility for these reduced titres could be a reduction in RNA replication within the bodies of AZT-treated mosquitoes and thus, a delay in dissemination and transmission. However, we did not observe significant differences in viral RNA by quantitative PCR with reverse transcription (qRT-PCR) in the bodies (Fig. 4e, bodies). Interestingly, we detected viral RNA in the legs and wings of AZT-treated mosquitoes (Fig. 4e; legs and wings) despite the absence of infectious viral particles (Fig. 4d; legs and wings). This suggests that while robust viral RNA replication was occurring in the presence of AZT, infectious particles were unable to reach the legs, wings and saliva early in infection. A possible explanation could be the release of viral RNA from the primary sites of infection as a consequence of cellular/tissue damage and a loss of tolerance, rather than resistance, to viral infection in the absence of vDNA.

Given the results from early time points during infection, we inquired whether viral titres continued to increase late in infection and specifically at the time of mosquitoes' death. We hypothesized that during this lethal phase of the infection, viral titres would be steadily increasing. We treated mosquitoes with 5 mg ml^−1^ of AZT, conditions under which the rapid death of mosquitoes reproducibly occurred 9 days post-infection. Under these conditions, we again found a reduction in viral transmission early in infection (day 3 saliva, Supplementary Fig. 8) as well as a slight reduction in viral titres in the bodies at day 11. Interestingly, although we did observe an increase in viral titres in the legs and wings at day nine post-infection, we found no striking differences during viral infection (bodies), dissemination (legs and wings) or transmission potential (saliva) at late time points (Fig. 4d and Supplementary Fig. 8). The results suggest that death was not due to an increased viral burden (loss of resistance), but was rather the consequence of a lack of tolerance for the arbovirus infection. Taken together, these data show that mosquitoes generate vDNA through an endogenous reverse transcriptase activity-dependent mechanism. Since the inhibition of vDNA production leads to the death of the infected mosquitoes without reducing viral titres, our data suggest that generation of vDNA in mosquitoes controls tolerance to arbovirus infection, rather than resistance as observed in *Drosophila*^26^.

## Discussion

This study provides insight into how mosquitoes become efficient arbovirus vectors, as they remain infected for their lifetime without succumbing to infection. We show that fragments of RNA viruses are reverse transcribed in insect hosts by an endogenous reverse transcriptase activity and that the resulting vDNA reinforces the RNAi response against viral infections. These results further extend our earlier discovery in *Drosophila*^26^ to another insect-virus system, suggesting that vDNA biogenesis could be a widespread phenomenon associated with RNA virus infections in insects, and linked to virus tolerance in mosquitoes. This conclusion is not only based on observations with CHIKV in *Ae. albopictus*, but also reinforced by the presence of vDNA in another species, *Ae. aegypti*, infected with dengue virus. Thus, our data suggest that vDNA has a crucial role in establishing viral tolerance, allowing vector survival and, consequently, the spread of arboviral pathogens^18^,^19^.

From a mechanistic perspective, vDNA formation appears to be an early requirement to mount an effective systemic immunity. The synthesis of vDNA molecules is dependent on pre-existing host reverse transcriptase activity, possibly originating from endogenous retrotransposons, both in infected cultured cells and mosquitoes. This ensures the rapid appearance of vDNA on infection, both in cultured cells and *in vivo*, within 6 h and 2 days, respectively, when infectious viral particle production is rapidly increasing. In addition to the presence of vDNA in bodies of infected mosquitoes, we also detected vDNA in the legs and wings during infection. Although it is possible that vDNA is formed *in situ* when legs and wings are in turn infected, an interesting possibility would be that vDNA circulates from the original infected zone and serves as a systemic immune signal to warn or prime uninfected cells, which impedes infection. We speculate that mosquitoes rely on active processes such as the assembly and egress of viral particles, cell lysis or cellular export pathways, to release vDNA, which could in turn act as a danger signal for uninfected cells. One can imagine that after the primary infection in the midgut, vDNA is produced and released (along with viral RNA) into the circulatory system. The vDNA might travel between tissues to carry antiviral information. While our results suggest that vDNA is a mobile signal, it is not incompatible with occasional genomic integration events. Complete and fully annotated reference genomes for *Ae. albopictus*^28^ and other insects will help solve this puzzle and broaden our observations to other insect vectors.

Our results show the contribution of vDNA in the production of virus-specific small RNAs during the antiviral immune response. However, we found contrasting results between cultured cells and living mosquitoes. In the time course of an infection in cultured cells, vDNA seems mostly to influence vpiRNA synthesis. In contrast, vpiRNAs are barely detected *in vivo*, and suppressing vDNA formation delays vsiRNA synthesis, consistent with a more prevalent vsiRNA, rather than vpiRNA, response in CHIKV-infected mosquitoes. In addition, the vsiRNAs were produced and detected earlier in infection than vpiRNAs, consistent with the early requirement for vDNA in establishing a systemic immune response. When characterizing vpiRNAs two important observations can be made: (i) at early time points, we only detected primary vpiRNAs (vpiRNA-like molecules of the expected size, yet lacking any ping-pong signature); (ii) in late infection, secondary vpiRNAs (with ping-pong signature and U1-A10 bias) appeared. These results suggest that primary piRNAs are possibly generated from viral RNA replication intermediates, whereas those late in infection are generated from both viral RNA and vDNA, similar to endogenous piRNAs. Nonetheless, this may imply a bimodal innate immune response in mosquitoes, where the early detection of vsiRNAs may be linked to the initiation of viral infection and the detection of vpiRNAs late in infection could be linked to the spread of viral particles to different organs and thus be a reflection of a tissue-specific RNAi immune response. Interestingly, a previous study using a West African lineage of CHIKV detected both vsiRNAs and vpiRNAs in infected *Ae. albopictus* mosquitoes at day 4 post-infection^6^. This difference could be related not only to the speed by which the virus reaches different organs, but also to the specific viral strain studied. Additional *in vivo* studies and the establishment of new insect models infected with their corresponding natural viruses could help understanding whether the antiviral response might be targeted towards specific viruses or tissues using different RNAi pathways.

Furthermore, these studies open the possibility of additional role(s) for vDNAs, vsiRNAs and vpiRNAs in the mosquito life cycle, with impacts on fertility, development, and both horizontal and vertical transmission of arboviruses. Finally, we note that the vital role of vDNAs in mosquito tolerance and arbovirus transmission raises the intriguing possibility that arboviruses might have evolved mechanisms to counteract vDNA synthesis or function, analogous to viral suppressors of RNAi in insect-only viruses^29^,^30^,^31^.

In conclusion, these studies bring to light the innate immune mechanisms used by mosquitoes to cope with arbovirus infections in nature. The rapid synthesis of vDNA molecules is essential for the synthesis of small RNA molecules and subsequent tolerance to viral infection, which renders the mosquito an efficient vector for transmission. In addition, these studies provide a rationale for a conceptually novel strategy for vector control. Historically, vector control has relied on the mass elimination of vectors regardless of their infection status. More recently, vector population replacement strategies have proposed to substitute susceptible vectors for insects rendered resistant to infection by means of genetic modification or transfection with bacterial endosymbionts^32^. Whereas replacement strategies have focused until now on increasing resistance to infection, our results open the way for a new class of replacement strategies that would act to decrease vector tolerance to infection. Similar to ‘evolution-proof' insecticides^33^, selective killing of a small fraction of the vector population would considerably delay resistance evolution. Moreover, it is tempting to speculate that lack of tolerance (loss of function) might be easier to achieve than resistance (gain of function) to infection. Although much work remains to be done before such a strategy can be implemented, evidence of mosquito death following arbovirus infection in the absence of vDNA provides the proof-of-principle that infected mosquitoes can be selectively eliminated.

## Methods

### Cell culture

*Aedes albopictus* (C6/36 (ATCC CRL-1660) and U4.4 (ref. 34)) and *Aedes aegypti* (Aag2^35^) cell lines were a kind gift from G.P. Piljman, Wageningen University, the Netherlands. Cell lines were maintained at 28 **°**C with 5% CO_2_ in L-15 Leibovitz medium (Gibco, UK) supplemented with 10% foetal bovine serum (Gibco, UK), 1% nonessential amino acids (Gibco, UK), 2% tryptose phosphate broth (Sigma) and 1% penicillin/streptomycin (P/S; Gibco, UK). BHK-21 and Vero cells were maintained at 37 **°**C with 5% CO_2_ in Dulbecco's modified eagle medium (DMEM) (Gibco, UK) supplemented with 10% foetal calf serum (Gibco, UK) and 1% P/S.

### Viruses

CHIKV from the Indian Ocean Lineage was generated from the previously described infectious clone^36^. To generate wild-type CHIKV, 10 μg of each plasmid was linearized overnight with *NotI* (Fermentas), purified by phenol:chloroform extraction and ethanol precipitation, and resuspended in nuclease-free H_2_O. Viral RNAs were *in vitro* transcribed using the SP6 mMessage mMachine kit (Ambion, Austin, TX, USA) following the manufacturer's instructions. RNAs were treated for 20 min with Turbo DNAse, purified by phenol:chloroform extraction and ethanol precipitation, and resuspended in nuclease-free H_2_O at a concentration of 1 μg μl^−1^ and stored at −80 °C. BHK-21 cells (ATCC CCL-10) were then electroporated with 10 μg of the *in vitro* transcribed infectious clone RNA. In brief, BHK cells were trypsinized (Gibco, UK), washed twice with ice-cold phosphate-buffered saline (PBS), and resuspended at a concentration of 2 × 10^7^ cells per ml in cold PBS. Cells were then electroporated at 1.2 kV, 25F, with infinite resistance in an XCell Gene Pulser (Bio-Rad, Hercules, CA). Cells were allowed to recover for 10 min at room temperature, added to pre-warmed media and incubated at 37 °C for 48 h. Viral containing supernatants were recovered, centrifuged at 1,200 r.p.m. for 5 min to remove cellular debris, and viruses were aliquoted and stored at −80 °C. Infectious viral titres were determined by plaque assay on Vero cells as described below.

This study used a dengue virus isolate belonging to serotype 1 (KDH0026A) derived from a human serum sample collected in 2010 from a clinically ill dengue patient attending Kamphaeng Phet Provincial Hospital, Thailand^37^. The isolate was passaged <5 times in C6/36 cells (ATCC CRL-1660) before experimental infections of mosquitoes.

### Virus titrations

Viral titres were determined by plaque assay on Vero cells (ATCC CRL-1586). Tenfold serial dilutions of each viral sample were made in DMEM and incubated on a monolayer of Vero cells for 1 h at 37 °C. Following incubation, cells were overlaid with 0.8% agarose (invitrogen) containing DMEM supplemented with 2% foetal calf serum and 1% P/S. For all mosquito samples, 1% antibiotic-antimycotic (Anti-Anti, Gibco, UK) was added. Cells were incubated for 72 h to allow plaques to develop. Cells were then fixed with 4% formalin, agarose plugs removed and plaques were visualized by the addition of crystal violet (10% crystal violet and 20% ethanol). Titres were determined on the highest dilution where plaques could be visualized and counted.

### RNA extractions and qRT-PCR

Virus containing samples were extracted with TRIzol (Invitrogen) following the manufacturer's instructions and resuspended in nuclease-free H_2_O. Before qRT-PCR analysis, all RNA samples were treated for 30 min with DNase I (Roche) and the DNase was inactivated by incubation at 75 °C for 30 min. qRT-PCR analysis was performed using the RNA-to-Ct kit (Applied Biosystems, Foster, CA, USA), the forward primer 5′-TCACTCCCTGCTGGACTTGATAGA-3′ and reverse primer 5′-TTGACGAACAGAGTTAGGAACATACC-3′, and FAM-labeled probe 5′-AGGTACGCGCTTCAAGTTCGGCG-3′ as previously described^36^. Each sample was run in duplicate and RNA was quantified using a purified *in vitro* transcribed RNA as a standard.

### vDNA PCR analysis

Each sample was homogenized in 50 μl of squishing buffer (100 mM Tris-HCl, 25 mM NaCl, 1 mM EDTA pH 8, 0.2 mg ml^−1^ proteinase K (eurobio)), incubated at 37 °C for 1 h, and Proteinase K was inactivated at 95 °C for 2 min. 1.5 μl of the treated sample was subjected to PCR. PCR analysis for detection of vDNA was performed using DreamTaq DNA polymerase (Thermo Scientific) with the CHIKV forward primer 5′-CACCGACGTGATGAGAC-3′ and reverse primer 5′-GATGCGGCTGCTGTCATGAC-3′, or forward primer 5′-TCACTCCCTGTTGGACTTGATAGA-3′ and reverse primer 5′-TTGACGAACAGAGTTAGGAACATACC-3′. For dengue virus forward primer 5′-ACCGAGCTGGGCCAGTGTCA-3′ and reverse primer 5′-TCCAGCCAGCGGGGTCGTAG-3′ were used. The housekeeping 18S rRNA gene was used with the forward primer 5′-GGTCGGCGCGGTCGTAGTGTGG-3′ and reverse primer 5′-TCCTGGTGGTGCCCTTCCGTCAAT-3′.

RT-PCR for detection of viral RNA was performed using 1 μg of total DNase treated RNA and SuperScript II reverse transcriptase (Invitrogen) following the manufacturer's protocol. 18S rRNA was used as control.

### *In vitro* reverse transcriptase assay

Insect cell extracts: Aag2, C6/36, U4.4 and S2 (Invitrogen R69007) cell pellets (10^7^ cells per reaction) were washed once with PBS and flash-frozen in liquid nitrogen. For the reactions, cells were lysed in CHAPS lysis buffer (10 mM Tris-HCl pH 7.5, 400 mM NaCl, 1 mM MgCl_2_, 1 mM EGTA, 0.5% CHAPS, 10% glycerol, freshly supplemented with complete EDTA-free protease inhibitors cocktail (Roche) and 1 mM DTT). After incubation at 4 °C for 10 min, cell debris were removed by centrifugation at 16,000*g* for 10 min at 4 °C. Supernatants were transferred to clean tubes. Total protein concentration was determined by Bradford assay (Bio-Rad).

Reverse transcriptase assays were carried out for 15 min at 25 °C in 50 μl reactions containing 4 μg of insect cell extracts, 320 ng of PAGE-purified oligo(dT)_20_ (Eurogentec), 500 ng of poly(rA) (GE Healthcare) and 1 μCi of α-^32^P-dTTP (3,000 Ci mmol^−1^, PerkinElmer) in 50 mM Tris-HCl (pH 7.5), 50 mM KCl, 5 mM MgCl_2_ or 0.7 mM MnCl_2_, 5 mM DTT and 0.1% Triton X-100. Then, 5 μl of each reaction were spotted in triplicate onto DE-81 paper; an ion exchange paper that retains incorporated nucleotides but not free dNTPs. Papers were washed 5 times with 100 ml of 2X saline-sodium citrate solution and exposed to a PhosphorImager screen. The nucleoside analogue and RT inhibitor AZTTP was obtained from eEnzyme. Reverse transcriptase assays with AZTTP were conducted similarly but in 25 μl reactions with twice less amount of each reagent. Note that we used AZTTP *in vitro* assays because it corresponds to the bioactive form of AZT.

### *In vitro* dsRNA silencing assay

3.5 × 10^4^ mosquito cells per well (C6/36, U4.4 or Aag2 cell lines) were seeded in a 96-well plate with different concentrations of AZT (Sigma), ranging from 0 to 10 mM. The day after cell were transfected with 12 ng pMT firefly luciferase, 6 ng pMT renilla luciferase and 100 ng dsRNA firefly luciferase, using Effectene (QIAgen) following the manufacturer's instructions. Next day cells were washed to remove the excess of plasmids and dsRNA. Forty-eight hours after transfection, plasmid expression was induced by addition of CuSO_4_ to the medium at a final concentration of 500 μM for 24 h. Luminiscence was measured using the Dual-Glo Luciferase Assay System (Promega).

### Viral growth curves and RNA synthesis

Virus was diluted to an MOI of 0.1 in L-15 media without serum and incubated with C6/36, U4.4, and Aag2 cells for 1 h at 28 °C. Cells were washed with PBS to remove unbound virus and complete media was added. Supernatant was harvested at 24 and 48 h and infectious titres were determined by plaque assay as described above. Viral RNA was extracted from the supernatant and quantified by qRT-PCR. Cells were then harvest and the vDNA was analysed as described previously.

### Mosquito infections and manipulations

A laboratory colony of *Ae. albopictus* was used for mosquito infections 8-13 generations after it was collected in 2011 in Phu Hoa, Ben Cat District, Binh Duong Province, Vietnam. Field-derived *Ae. aegypti* mosquitoes originally collected in Nakhon Chum, Muang District, Kamphaeng Phet Province, Thailand were used for experimental infections within three generations of laboratory colonization.

Two days before mosquito infection, adult mosquitoes were either left to feed on 10% sucrose water (untreated) or 10% sucrose water containing either 5 or 10 mg ml^−1^ AZT (Sigma, St Louis, MO, USA). On the day of infection, 7-day-old female mosquitoes were allowed to feed on pre-washed rabbit blood meals containing 5 mM ATP, 10^6^ PFU ml^−1^ CHIKV, and either 10% sucrose or 5 or 10 mg ml^−1^ AZT for 30 min at 37 °C. After blood feeding, engorged females were sorted into individual boxes (20 mosquitoes/box) and incubated at 28 °C with 70% humidity. Mosquitoes were fed on either 10% sucrose or 10% sucrose containing AZT (5 or 10 mg ml^−1^) for the duration of the experiment. At the end of incubation, legs and wings were removed, and saliva was collected from individual mosquitoes by inserting the proboscis into a capillary tube containing 5 μl of foetal bovine serum for 45 min at room temperature. The saliva was collected in 45 μl of PBS and the body and legs and wings were placed into 2 ml round bottom tubes containing 200 μl of PBS and a steel ball (Qiagen, Chatsworth, CA, USA). Bodies, as well as legs and wings, were ground in a MM300 homogenizer (Qiagen, Chatsworth, CA, USA) at 30 shakes per s for 2 min. Viral titres were determined by plaque assay and vDNA and RNA levels were analysed as described above. Because viral titres and RNA levels were strongly non-normally distributed, differences between AZT treated and untreated individuals were assessed at each time point by the nonparametric Wilcoxon rank-sum test.

### Mosquito survival curves

Mosquito mortality at day 1 was attributed to damage invoked by feeding/selection procedure, and excluded from further analyses. Mortality was monitored daily for 14 days, and every day the mosquitoes were fed with fresh 10% sucrose or AZT in 10% sucrose. In all experiments at least 40 mosquitoes per group were monitored. Each condition was repeated at least two times. Curves were analysed using Prism6 software (GraphPad *Software*, La Jolla, CA, USA).

### Small RNA libraries

Total RNA from mosquito cells or from pools of five whole untreated or AZT-treated CHIKV-infected mosquitoes was isolated with TRIzol (invitrogen). 19–33 nts length small RNAs were purified from a 15% acrylamide/bisacrylamide (37.5:1), 7 M urea gel as described in Gausson *et al.*^38^. Purified RNAs were used for library preparation using the kit NEBNext Multiplex Small RNA Library Prep for Illumina (E7300 L) with the 3′ adaptor from IDT (linker 1) and in-house designed indexed primers. Libraries were diluted to 4 nM and sequenced using NextSeq 500 High Output Kit v2 (75 cycles) on a NextSeq 500 (Illumina, San Diego, CA, USA). Sequence reads were analysed with in-house Perl scripts.

### Bioinformatics analysis of small RNA libraries

The quality of fastq files was assessed using graphs generated by ‘FastQC' (http://www.bioinformatics.babraham.ac.uk/projects/fastqc/). Quality and adaptors were trimmed from each read using ‘cutadapt' (https://code.google.com/p/cutadapt/). Only reads with acceptable quality (phred score ≥20) were retained. A second set of graphs was generated by ‘FastQC' (http://www.bioinformatics.babraham.ac.uk/projects/fastqc/) on the fastq files create by ‘cutadapt'^39^. Mapping was produced by ‘bowtie1'^40^ with the ‘-v 1' (one mismatch between the read and its target). CHIKV reference genome was from the infectious clone described above^36^. ‘bowtie1' generates results in ‘sam' format. All ‘sam' files were analysed by different tools of the package ‘samtools'^41^ to produce ‘bam' indexed files. To analyse these ‘bam' files, different kind of graphs were generated using home-made R scripts with several Bioconductor libraries as ‘Rsamtools' or ‘Shortreads' (http://bioconductor.org/).

### Data availability

Sequence data have been deposited in the Sequence Read Archive with accession code SRP062828. The authors declare that all other data supporting the findings of this study are available within the article and its Supplementary Information Files, or from the corresponding author upon request. In-house Perl scripts and R scripts are available from the corresponding author upon request.

## Additional information

**How to cite this article:** Goic, B. *et al.* Virus-derived DNA drives mosquito vector tolerance to arboviral infection. *Nat. Commun.* 7:12410 doi: 10.1038/ncomms12410 (2016).

## Supplementary Material

Supplementary Information Supplementary Figures 1-10
