# Supplementary material for: Virus-derived DNA drives mosquito vector tolerance to arboviral infection
Source: Nat Commun. 2016 Sep 1;7:12410. doi: 10.1038/ncomms12410 (PMC5025746; doi:10.1038/ncomms12410)
Supplement: Supplementary Information — Supplementary Figures 1-10 [file ncomms12410-s1.pdf]

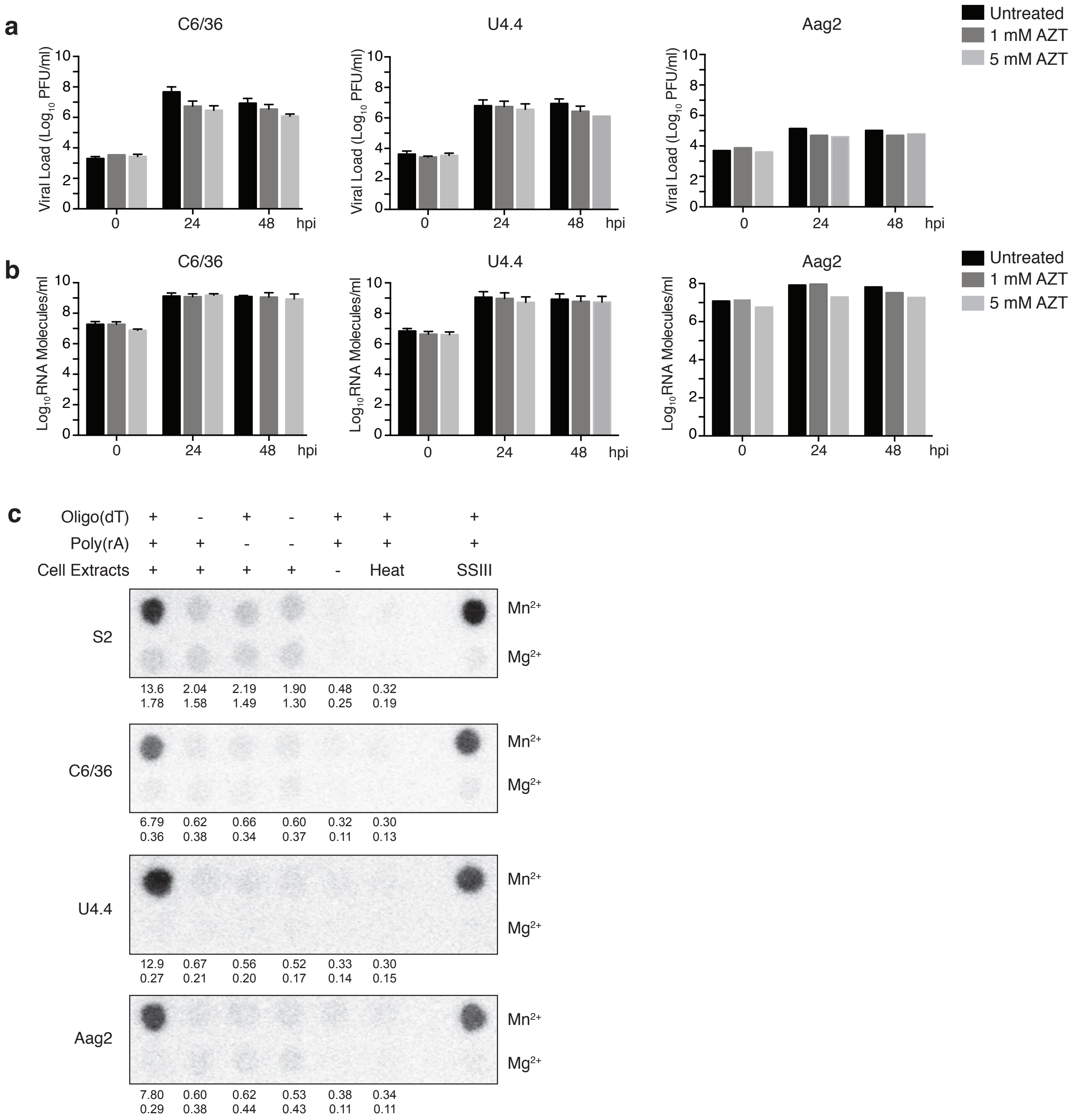


**Supplementary Figure 1: CHIKV growth and replication activity in the presence of AZT**. C6/36, U4.4, and Aag2 cells were infected at a MOI of 0.1 in the presence of 0, 1 or 5 mM of AZT. Cell culture supernatants were harvested at 0, 24 and 48 hours post infection (hpi). **(a)** Viral titers were determined by plaque assay on Vero cells. **(b)** Viral RNA levels were determined by qRT-PCR. Viral growth was preformed in triplicate. Error bars represent the standard deviation. **(c)** Endogenous reverse-transcriptase activity of S2 (control), C6/36, U4.4, and Aag2 cell extracts, Superscript III (SSIII, used in place of insect cell extracts) or heat-inactivated cells (Heat), assessed in the presence of $Mn^{2+}$ or $Mg^{2+}$ and various combinations of oligo(dT) and poly(rA) (indicated above each image), spotted onto DE81 paper (which retains poly(dT) products but not free [$^{32}$P]dTTP) and measured at initial velocity phase. Below each image, quantification of the spots, in arbitrary units. A Student's t-test was used to determine statistical significance. Absence of P-value indicates a non-significant.

**Goic *et al.*, Supplementary Figure 1**

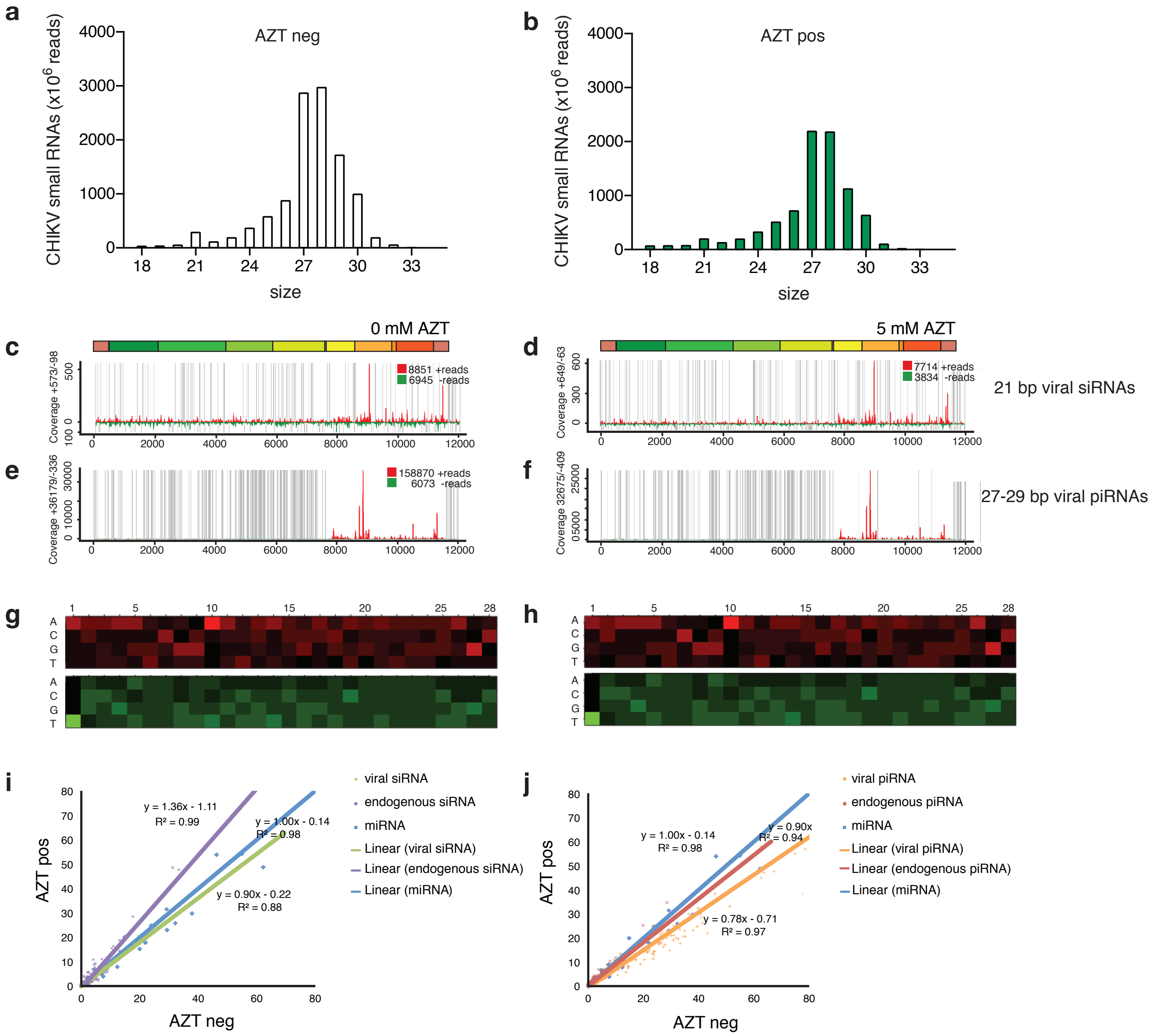


**Supplementary Figure 2: Chikungunya vDNA contributes to the production of vpiRNAs in C6/36 cells.** Infected C6/36 cells were **(a)** left untreated (clear bars) or **(b)** treated with 5 mM AZT (green bars) for three days. Graph represents the size distribution of the total number of CHIKV specific small RNA reads (ranging from 18 to 33 nts) normalized to the total number of reads. **(c-d)** Very low but homogeneous coverage of vsiRNAs in C6/36 cells. The coverage of vpiRNAs on the CHIKV genome in **(e)** untreated cells or **(f)** AZT-treated cells. Most of the CHIKV vpiRNA reads belong to the 3'-terminal region on the viral genome. The sense and anti-sense small RNAs are in red and green, respectively. Grey lines represent uncovered regions. **(g-h)** Relative nucleotide frequency per position of the 27-29 nt viral small RNAs that map to the sense and anti-sense strand of the viral genome, red and green respectively. The intensity varied in correlation with the frequency. A nucleotide bias (U1 and A10) is observed. **(i-j)** Accumulation of viral and cellular small RNAs in C6/36 cell line three days post CHIKV infection in the presence (AZT pos) or absence (AZT neg) of AZT, assessed as **(i)** mapping of small RNA corresponding to CHIKV vsiRNA (green), endo-siRNA (purple) belonging to the gene *GAPW01000199* or to miRNA (blue). For miRNA each dot represents one miRNA. For siRNAs each dot represents the coverage of a region of 20 bases of the target RNA. The lines for miRNA and endo-siRNA are superposed. **(j)** Mapping of small RNA corresponding to CHIKV vpiRNA (orange), endo-piRNA (red) belonging to the gene *GAPW01000199* or to miRNA (blue). For miRNAs each dot represents one miRNA. For piRNAs each dot represents the coverage of a region of 20 bases of the target RNA. For **(h-i)** lines represent the linear trendline of each set of values. The equation and R-squared value of each regression are also men-

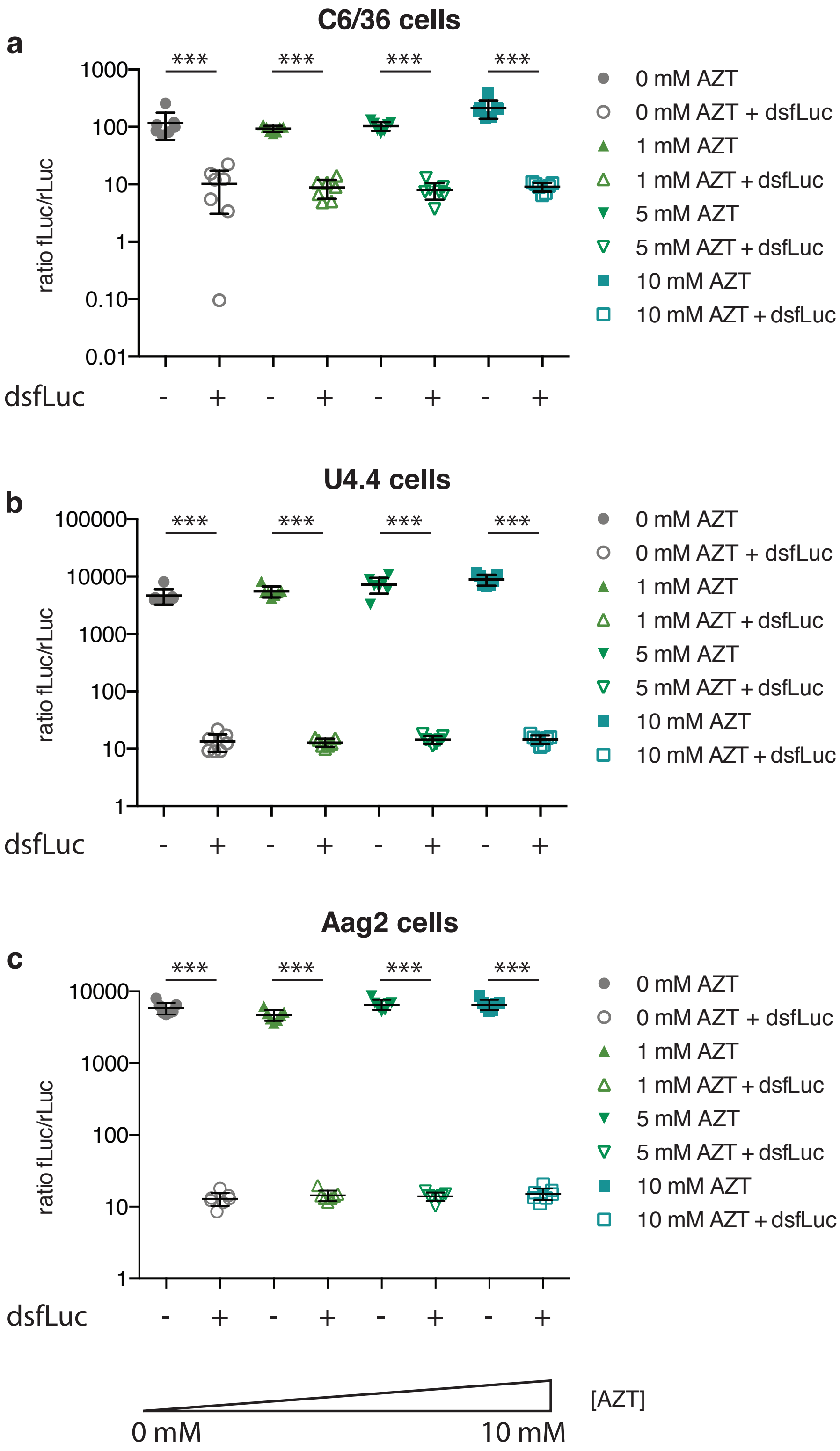


**Supplementary Figure 3: AZT treatment does not impair the RNAi machinery.** Mosquito cell lines were co-transfected with plasmids expressing firefly luciferase (fLuc), renilla luciferase (rLuc) and dsRNA carrying firefly luciferase sequence (dsfLuc). Renilla luciferase was used to normalize. Cells were treated with different doses of AZT (ranging from 0 to 10 mM) for 48 hours. After 24 hours post induction luciferase activity was measured in **(a)** C6/36 cell line, **(b)** U4.4 cell line and **(c)** Aag2 cell line. Each experiment was completed at least two times, with 8 replicates each. Error bars represent the standard deviation. A pair-wise comparison based on a Mann-Whitney analysis was used to determine statistical significance (***$P < 0.001$).

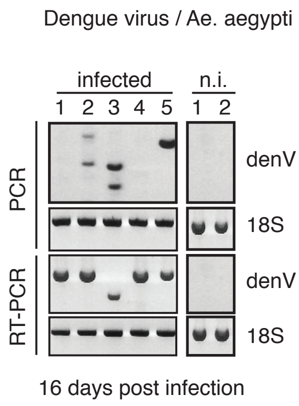


**Supplementary Figure 4: Dengue vDNA is present in infected *Aedes aegypti* mosquitoes.** *Ae. aegypti* mosquitoes were infected with dengue virus by artificial blood meal. Sixteen days post infection mosquitoes were analyzed for dengue vDNA. Non-infected (n.i.) mosquitoes were used as negative controls. 18S was used as a housekeeping gene loading control.

**Goic *et al.*, Supplementary Figure 4**

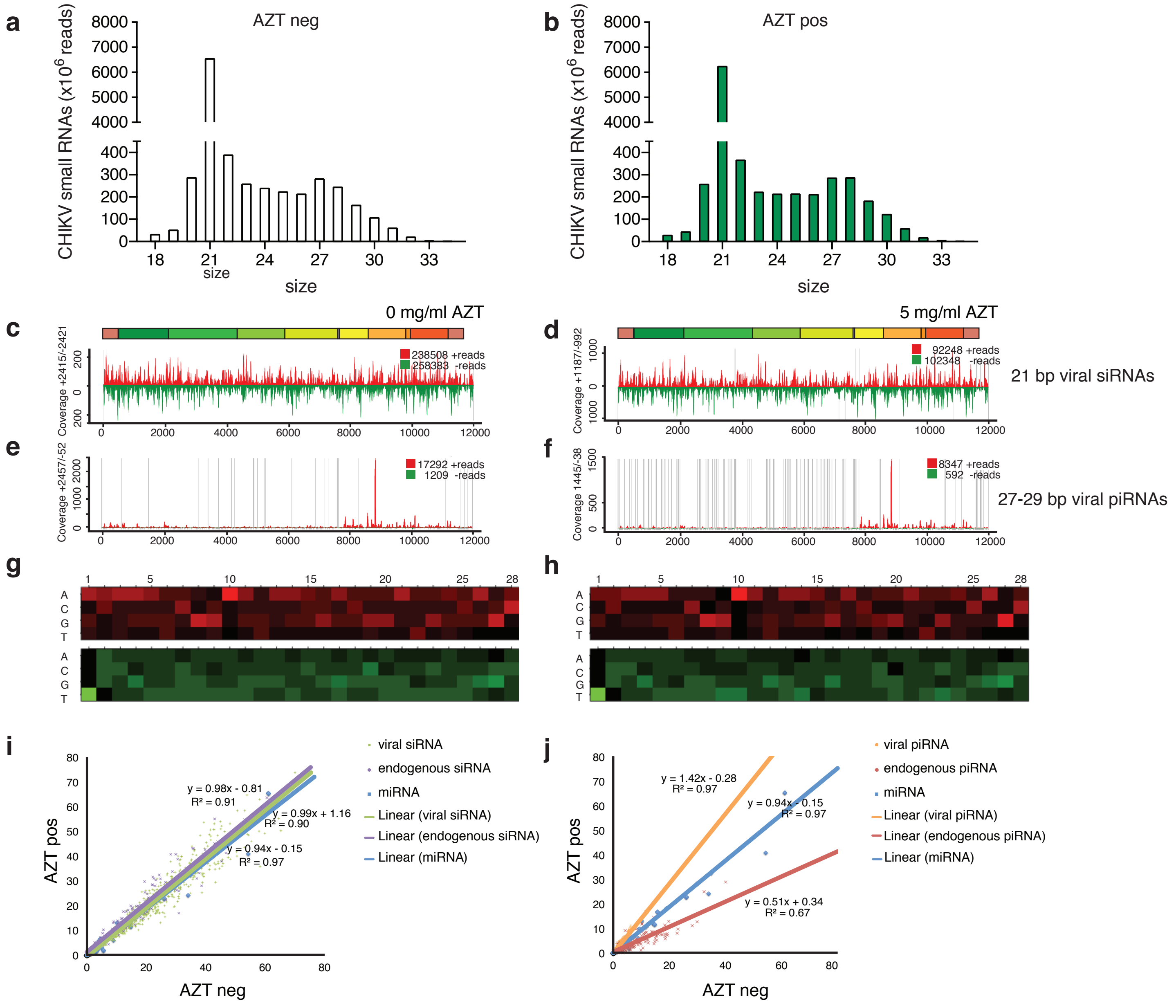


**Supplementary Figure 5: Chikungunya virus vDNA does not contribute to the production of vsiRNAs late in infection *in vivo*.** Small RNA libraries of CHIKV infected mosquitoes. **(a)** *Ae. albopictus* mosquitoes were untreated (clear bars) or **(a)** treated with 5 mg/ml AZT (green bars) and harvested 9 dpi. Five whole mosquitoes were pooled from each condition for the generation of small RNA libraries. Graphs represent the size distribution of the total number of CHIKV specific small RNA reads (corresponding to the positive and negative strand orientation of the viral genome) ranging from 18 to 33 nts normalized by the total number of reads. **(c-f)** Panels show the coverage of CHIKV genome at 9 dpi using the 21 or 28 nts long small RNAs at the different conditions (untreated: c and e or AZT-treated: d and f). The sense and anti-sense small RNAs are in red and green, respectively. Grey lines represent uncovered regions. **(g-h)** Relative nucleotide frequency per position of the 27-29 nt viral small RNAs that map to the sense and anti-sense strands of the viral genome, red and green respectively. The intensity varied in correlation with the frequency. A nucleotide bias (U1 and A10) is observed. **(i-j)** Accumulation of viral small and cellular RNAs in *Ae. albopictus* nine days post CHIKV infection in the presence (AZT pos) or absence (AZT neg) of AZT, assessed as **(i)** mapping of small RNA corresponding to CHIKV vsiRNA (green), endo-siRNA (purple) belonging to the gene *GAPW01000199* or to miRNA (blue). For miRNA each dot represents one miRNA. For siRNAs each dot represents the coverage of a region of 20 bases of the target RNA. The lines for miRNA and endo-siRNA are superposed. **(j)** Mapping of small RNA corresponding to CHIKV vpiRNA (orange), endo-piRNA (red) belonging to the gene *GAPW01000199* or to miRNA (blue). For miRNAs each dot represents one miRNA. For piRNAs each dot represents the coverage of a region of 20 bases of the target RNA. For **(i-j)** lines represent the linear trendline of each set of values. The equation and R-squared value of each regression are also mentioned.

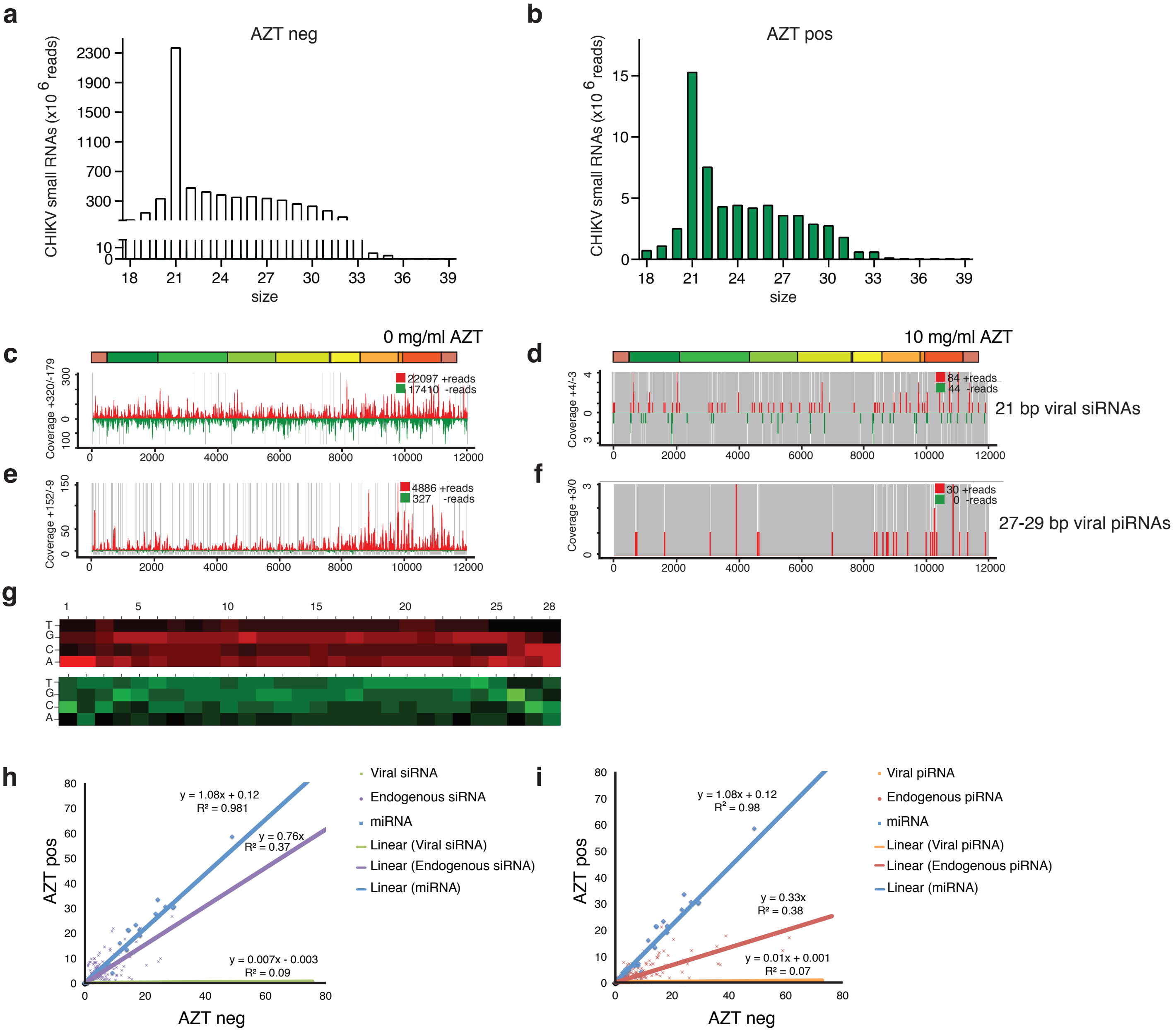


**Supplementary Figure 6: AZT inhibition of chikungunya virus vDNA and vsiRNAs is dose-dependent *in vivo*.** Small RNA libraries of CHIKV infected mosquitoes. **(a)** *Ae. albopictus* mosquitoes were untreated (clear bars) or **(b)** treated with 10 mg/ml AZT (green bars) and harvested 3 dpi. Five whole mosquitoes were pooled from each condition for the generation of small RNA libraries. Graphs represent the size distribution of the total number of CHIKV specific small RNA reads (corresponding to the positive and negative strand orientation of the viral genome) ranging from 18 to 33 nts normalized by the total number of reads. **(c-f)** Panels show the coverage of CHIKV genome at 3 dpi using the 21 or 28 nts long small RNAs at the different conditions (untreated: c and e or AZT-treated: d and f). The sense and anti-sense small RNAs are in red and green, respectively. Grey lines represent uncovered regions. **(g)** Relative nucleotide frequency per position of the 27-29 nt viral small RNAs that map to the sense and anti-sense strands of the viral genome, red and green respectively. The intensity varied in correlation with the frequency. No nucleotide bias (U1 and A10) is observed. For AZT treated mosquitoes, the absence of negative strand reads, precludes the detection of ping-pong signature. **(h-i)** Accumulation of viral and cellular small RNAs in *Ae. albopictus* three days post CHIKV infection in the presence (AZT pos) or absence (AZT neg) of AZT, assessed as **(h)** mapping of small RNA corresponding to CHIKV vsiRNA (green), endo-siRNA (purple) belonging to the gene *GAPW01000199* or to miRNA (blue). For miRNA each dot represents one miRNA. For siRNAs each dot represents the coverage of a region of 20 bases of the target RNA. The lines for miRNA and endo-siRNA are superposed. **(i)** Mapping of small RNA corresponding to CHIKV vpiRNA (orange), endo-piRNA (red) belonging to the gene *GAPW01000199* or to miRNA (blue). For miRNAs each dot represents one miRNA. For piRNAs each dot represents the coverage of a region of 20 bases of the target RNA. For **(h-i)** lines represent the linear trendline of each set of values. The equation and R-squared value of each regression are also mentioned.

**a**

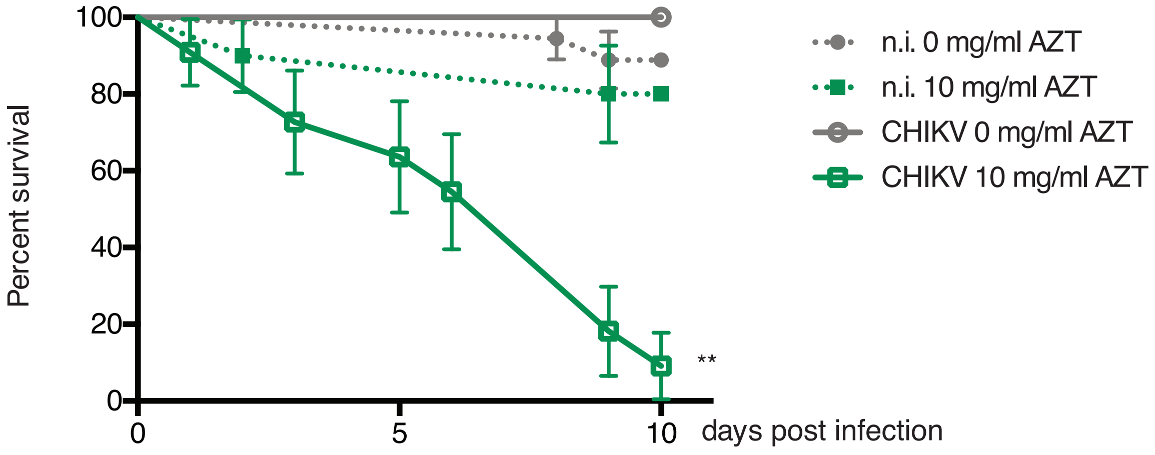


**b**

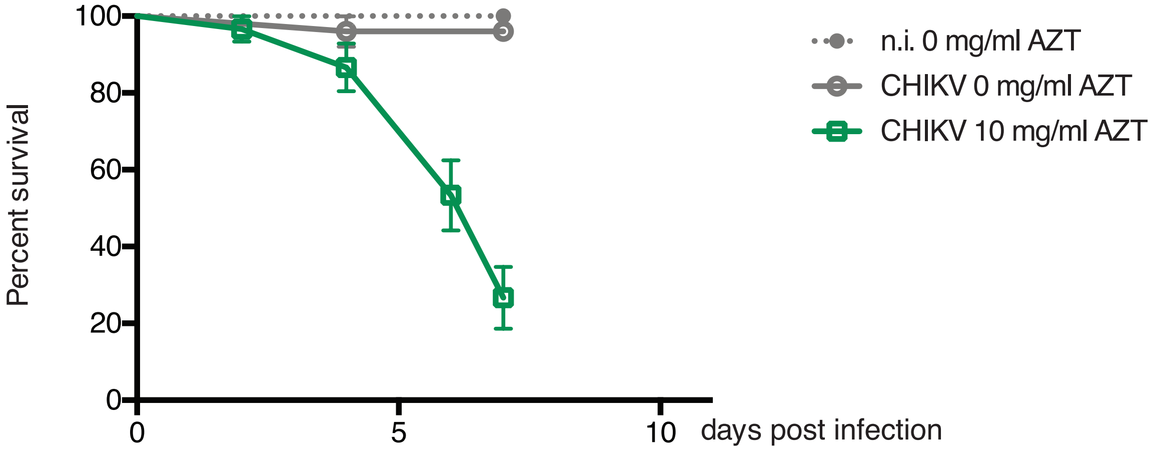


**Supplementary Figure 7: Mosquito survival curves in the presence or absence of AZT. (a-b)** Two independent survival curves of CHIKV infected mosquitoes treated daily with 0 or 10 mg/ml of AZT. After the infectious blood meal survival of mosquitoes was monitored daily for **(a)** 10 or **(b)** 7 days. Continuous lines show the lifespan of AZT-treated and infected mosquitoes while dotted lines show lifespan of AZT-treated but non-infected (n.i.) mosquitoes. Grey lines: 0 mg/ml AZT and green lines: 10 mg/ml AZT. For each curve at least 40 mosquitoes were used per condition. Error bars represent the standard deviation. P-values were calculated using a Gehan-Breslow-Wilcoxon test using noninfected mosquitoes with the same AZT treatment as a reference (**$P < 0.01$). Absence of P-value indicates non-statistical significance.

**Goic *et al.*, Supplementary Figure 7**

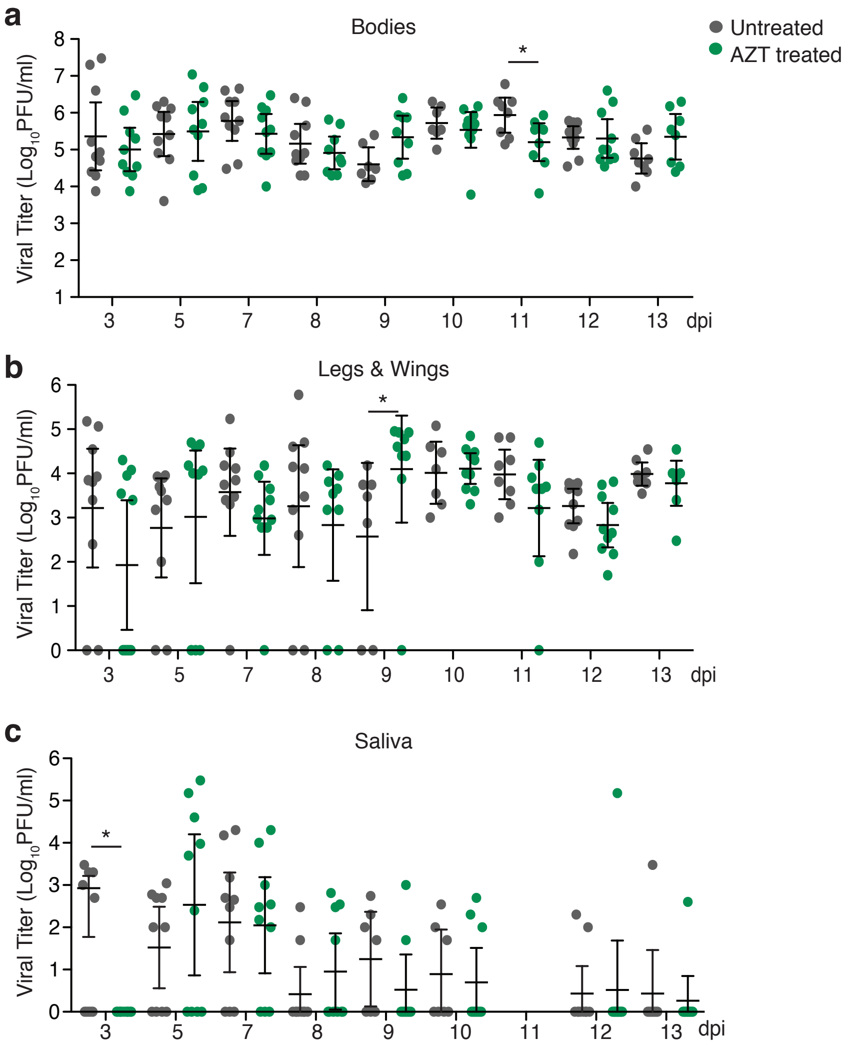


**Supplementary Figure 8: Viral titers of individual mosquitoes late during infection.** Viral titers of individual mosquitoes either untreated (grey dots) or treated with 5 mg/ml AZT (green dots) were determined over 14 days in **(a)** bodies, **(b)** legs & wings and **(c)** saliva by plaque assay. Standard deviation and geometric mean are shown. At least 10 mosquitoes were used per time point and condition. The experiment was completed two times. Error bars represent the standard deviation. A Wilcoxon rank-sum test was used to determine statistical significance (*P < 0.05). Absence of P-value represents non-statistical significance.

**Goic *et al.*, Supplementary Figure 8**

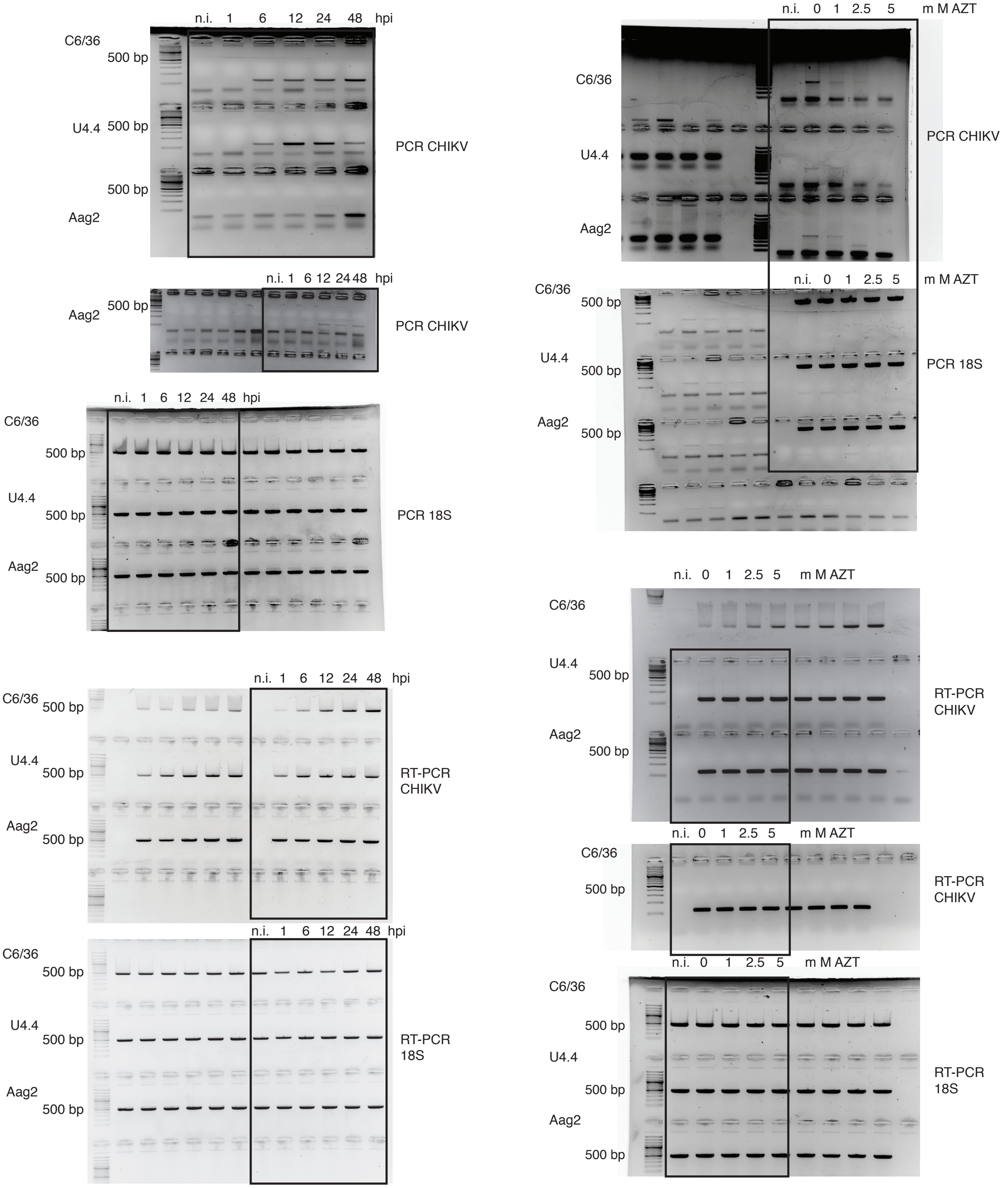


**Supplementary Figure 9:** Full gels of Fig. 1b (left column) and 1c (right column). Boxes indicate portion of gel used in primary figure.

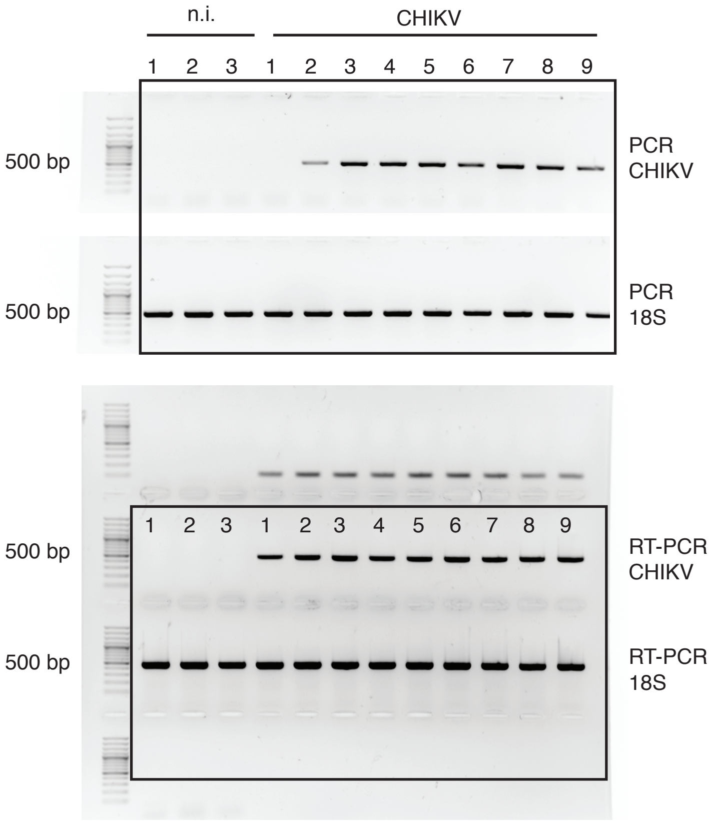


**Supplementary Figure 10:** Full gels of Fig. 3a. Boxes indicate portion of gel used in primary figure.
